# Cigarette smoking is associated with Herpesviruses in persons with and without serious mental illness

**DOI:** 10.1371/journal.pone.0280443

**Published:** 2023-01-18

**Authors:** Faith Dickerson, Emily Katsafanas, Andrea Origoni, Theresa Newman, Kelly Rowe, Rita S. Ziemann, Kamal Bhatia, Emily Severance, Glen Ford, Robert Yolken

**Affiliations:** 1 The Stanley Research Program at Sheppard Pratt, Baltimore, Maryland, United States of America; 2 The Stanley Neurovirology Laboratory, Department of Pediatrics, Johns Hopkins School of Medicine, Baltimore, Maryland, United States of America; 3 VanPelt Biosciences, Rockville, Maryland, United States of America; University of Illinois at Chicago, UNITED STATES

## Abstract

**Introduction:**

Herpesviruses are recognized as major causes of human diseases. Following initial infection, Herpesviruses can undergo cycles of reactivation controlled largely by the immune system. Cigarette smoking is an important modulator of the immune system particularly in individuals with serious mental illness where smoking is associated with increased rates of cardiopulmonary diseases and mortality. However, the effect of smoking on Herpesviruses has not been extensively studied.

**Methods:**

In this nested cohort study, cigarette smoking was assessed in 1323 persons with serious mental illness or without a psychiatric disorder ascertained in a psychiatric health care system and the adjacent community. Participants provided a blood sample from which were measured IgG class antibodies to five human Herpesviruses: Cytomegalovirus (CMV), Epstein Barr Virus (EBV), Herpes Simplex Virus-Type 1 (HSV-1); Varicella Zoster Virus (VZV); and Human Herpes Virus-Type 6 (HHV-6). The associations between smoking variables and antibody levels to the Herpesviruses were analyzed among diagnostic groups in multiple regression models adjusted for age, sex, and race.

**Results:**

Current smoking was significantly associated with higher levels of antibodies to CMV (coefficient .183, 95% CI .049, .317, p<.001, q<.007) and the three EBV proteins (EBV NA –(coefficient .088, 95% CI .032, .143, p = .002, q<.014; EBV Virion – coefficient .100, 95% CI .037, .163, p = .002, q<.014; and EBV VCA – coefficient .119, 95% CI .061, .177, p = .00004, q<.0016). The amount of cigarettes smoked was also correlated with higher levels of antibodies to the three EBV proteins. Interaction analyses indicated that the association between cigarette smoking and levels of antibodies to CMV and EBV was independent of diagnostic group. Cigarette smoking was not significantly associated with the level of antibodies to HSV-1, VZV, or HHV-6.

**Conclusions:**

Individuals who smoke cigarettes have increased levels of IgG antibodies to CMV and EBV. Cigarette smoking may be a contributory factor in the relationship between CMV, EBV and chronic somatic disorders associated with these viruses.

## Introduction

Human Herpesviruses are a diverse set of enveloped DNA viruses with worldwide prevalence. Following primary infection, which usually occurs before adulthood, human Herpesviruses can establish latency and subsequently reactivate throughout adult life. Past exposure to Herpesviruses and reactivation can be ascertained by the presence of viral specific IgG class antibodies in the blood. Herpesviruses are widely recognized as causes of serious infections in immunocompromised individuals such as those with HIV infection or those receiving immunosuppressive or anti-neoplastic medications. Herpesviruses are also being recognized as contributing factors to chronic disorders in immunocompetent individuals. Numerous studies have documented increased levels of exposure to Herpesviruses in individuals with atherosclerosis, pulmonary fibrosis, autoimmune disorders such as systemic lupus and multiple sclerosis, cognitive decline in the elderly, and several forms of cancer [1–3].

The outcome of infections with Herpesviruses is highly related to interactions with the host immune system. Cigarette smoking is recognized as one of the most important environmental factors which can alter immune functioning through interference with many immune pathways [4]. However, the effect of smoking on the rate of Herpesvirus infection has not been extensively studied.

The possible relationship between cigarette smoking and Herpesvirus infection is particularly concerning in individuals with serious psychiatric disorders since these individuals have increased rates of cigarette smoking as well as altered immune systems [5]. In previous studies of individuals with schizophrenia and other serious psychiatric disorders, we have documented that exposure to Herpesviruses such as Herpes Simplex Virus type 1 (HSV-1), Cytomegalovirus (CMV), and Epstein Barr Virus (EBV), as evidenced by increased levels of virus-specific IgG antibodies in the blood, can be associated with increased levels of cognitive impairments and excess mortality [6–8].

The goal of the current study was to define the association between smoking status and levels of antibodies to several Herpesviruses in a cohort of individuals with schizophrenia, bipolar disorder, major depressive disorder, and without a psychiatric disorder.

## Methods

### Study participants

Participants were individuals diagnosed with schizophrenia, bipolar disorder, major depressive disorder, or persons without a psychiatric disorder Who were enrolled in the Stanley Research Program at Sheppard Pratt in Baltimore, MD USA for a study of the association between infection, immunity, and psychiatric disorders. The participants with schizophrenia, bipolar disorder, and the non-psychiatric comparison group were enrolled between January 1, 2008, and December 31, 2021, and the participants with major depression in the period between March 1, 2013, and December 31, 2021.

The inclusion criterion for individuals with schizophrenia was a diagnosis of schizophrenia, schizophreniform disorder, or schizoaffective disorder. The inclusion criterion for individuals with bipolar disorder was a diagnosis of bipolar disorder including bipolar I disorder, bipolar II disorder, or bipolar disorder not otherwise specified. Those with major depressive disorder had either a single or recurrent episode. The psychiatric participants were recruited from inpatient and day hospital programs of Sheppard Pratt and from affiliated psychiatric programs. The diagnosis of each psychiatric participant was established by the research team including a board-certified psychiatrist and was based on the Structured Clinical Interview for DSM-IV Axis 1 Disorders and available medical records.

The inclusion criterion for the individuals without a psychiatric disorder was the absence of a current or past psychiatric disorder as determined by screening with the DSM-IV Axis I Disorders, Non-patient Edition. Persons in this group were recruited from posted announcements at local health facilities and universities in the same geographic area where the psychiatric participants were recruited.

Participants in all groups met the following additional criteria: age 18-65 (except the non-psychiatric controls who were aged 20-60); proficient in English; absence of any history of intravenous substance abuse; absence of intellectual disability by history; absence of HIV-1 infection; absence of serious medical disorder that would affect cognitive functioning; absence of a primary diagnosis of substance use disorder within the past three months per DSM-IV criteria.

The studies were approved by the Institutional Review Boards of the Sheppard Pratt and the Johns Hopkins Medical Institutions following established guidelines. IRB approval was obtained before study recruitment. All participants provided written informed consent after the study procedures were explained. Competence to give consent was assessed by research staff in the form of a short quiz following review of the written informed consent document; this method was approved by the Sheppard Pratt IRB.

### Demographic and clinical measures

Demographic and background information including self-reported race was obtained by interview during the study visit during which the blood sample was drawn as previously described [9]. Participants were asked if they were a current cigarette smoker and, if so, how many cigarettes or packs they smoked on a typical day (ppd). We employed the level of parental education as a marker of socioeconomic status. This was generally determined from the level of maternal education with the level of paternal education being employed when maternal education was not available. The level of parental education was characterized as < = 8 years, > 8 < = 12 years, >12< = 16 years, and >16 years.

### Antibody measurements

We employed solid phase immunoassays to measure IgG class antibodies to human Herpesviruses in blood samples obtained at study enrollment. These immunoassays were employed to measure IgG class antibodies to Cytomegalovirus (CMV), Epstein Barr Virus (EBV), Herpes Simplex Virus Type 1 (HSV-1), Varicella Zoster Virus (VZV) and Human Herpes Virus type 6 (HHV-6). In the case of EBV, IgG class antibodies were measured to three different antigens: virion antigen (EBV-virion) as well as the nuclear antigen (EBV-NA) and viral capsid antigen (EBV-VCA). All of the assays except for EBV-virion were performed using reagents which were commercially available, generally from IBL America, Minneapolis, Minnesota [7]. The EBV-virion assay was performed using purified EBV virions as previously described [10]. The values obtained from the immunoassays were measured in optical density units and normalized across different targets and solid phase media employing standardized scores based on mean and standard deviation also as previously described.

### Statistical analyses

Associations between quantitative antibody levels and smoking status were determined by linear regression models employing age, sex, race (white vs. non-white), and psychiatric diagnosis as covariates. Associations between antibody levels and the quantity of daily cigarette consumption were determined by means of linear regression and ordered logistic regression models employing the same covariates. For the purposes of analysis, the amount of smoking was characterized as none, >0 and less than .5 ppd, >.5 and less than 1 ppd, and > = 1 ppd. These models included interaction variables to determine potential interactions between cigarette smoking and clinical diagnosis. The variance inflation factor (VIF) was calculated for each regression analysis to determine collinearity. A value < = 2.5 was considered to represent a level of collinearity which did not significantly affect the outcome of the regression [11]. The relationship between pack years and levels of EBV antibodies were further explored by the measurement of marginal effects, defined as the partial derivative of the regression equation with respect to the outcome variable [12]. All statistical analyses were performed with STATA version 15, College Station, Texas. In addition to p values derived from the models, q values were calculated based on multiple comparisons using the Bonferroni correction. An association was considered to be significant if q< = .05.

## Results

The demographic and clinical characteristics of the 1323 individuals are shown in **Table 1.** The mean age at the time of assessment and sample collection was 35.0 years (s.d.12.8). A total of 612 (46%) participants were male and 740 (56%) were White. The diagnostic breakdown of the sample was schizophrenia, 468 (35.4%); bipolar disorder; 390 (29.5%); major depressive disorder, 124 (9.4%); and individuals without a psychiatric disorder, 341 (25.8%). A total of 489 (37.0%) individuals indicated that they smoked cigarettes at the time of evaluation. The rate of current cigarette smoking was particularly high in the individuals with schizophrenia (60.7%) as compared to the control group (13.5%) as shown in **Table 2**. The mean, standard deviation, and range of the antibody measurements in raw optical density units is depicted in S1 Table.

**Table 1:** Characteristics of study participants^1^.

	Tobacco Smokers	Non Smokers	Total
	N = 489	N = 834	N = 1323
Age ^2^	37.7 ± 12.6	33.5 ± 12.7	35.0 ± 12.8
Male ^3^	283 (57.9%)	329 (39.5%)	612 (46.3%)
Female	206 (42.1%)	505 (60.5%)	711 (53.7%)
Parental education ^4^			
< 8 years	36 (7.6%)	38 (4.6%)	74 (5.7%)
8-12 years	256 (53.7%)	332 (40.2%)	588 (45.1%)
12-16 years	143 (30.0%)	338 (40.9%)	481 (36.9%)
>16 years	42 (8.8%)	110 (14.4%)	161 (12.4%)
Race ^5^			
White	262 (53.6%)	478 (57.3%)	740 (55.9%)
Black	207 (42.3%)	252 (30.2%)	459 (34.7%)
Other	20 (4.1%)	104 (12.5%)	124 (9.4%)
Psychiatric Diagnosis ^6^			
Schizophrenia	282	186	468
Bipolar Disorder	137	253	390
Major Depressive Disorder	24	100	124
No psychiatric diagnosis	46	295	341
Packs Smoked per Day ^7^			
0		834 (64.1%)	
>0 and < = 0.5	260 (20.0%)		
>0.5 and < = 1.0	151 (11.6%)		
>1.0	57 (4.4%)		

^1^ Mean and standard deviation for continuous variables and number; percentage for dichotomous variables

**^2^** F = 34.02 (2), p<.001

^3^ chi2 = 42.10; p<.001

^4^ chi2 = 34.29; p<.001. Parental education was generally determined from the level of maternal education with the level of paternal education employed when maternal education was not available. Information on parental education was available from 1304 of the 1323 participants.

^5^ chi2 = 41.5, p<.001

^6^ chi2 = 206.69, p<.001

^7^ n = 1302

**Table 2:** Cigarette smoking status and packs per day smoked by diagnostic group.

	Schizophrenia	Bipolar Disorder	Major Depressive Disorder	Non-psychiatric Group	Total
	N = 468	N = 390	N = 124	N = 341	N = 1323
Current Cigarette Smoker	282 (60%)	137 (35%)	24 (19%)	46 (13%)	489 (37%)
Packs Smoked per Day^1^					
None	186 (40%)	253 (66%)	107 (87%)	295 (87%)	841 (64%)
>0 and < = 0.5	131 (28%)	76 (20%)	13 (11%)	40 (12%)	260 (20%)
>0.5 and < = 1.0	104 (22%)	39 (10%)	2 (2%)	6 (2%)	151 (12%)
>1.0	42 (9%)	14 (4%)	1 (1%)	0	57 (4%)

^1^ Schizophrenia n = 463, Bipolar Disorder n = 382, Major Depressive Disorder n = 123, total N = 1309

**Fig 1:**
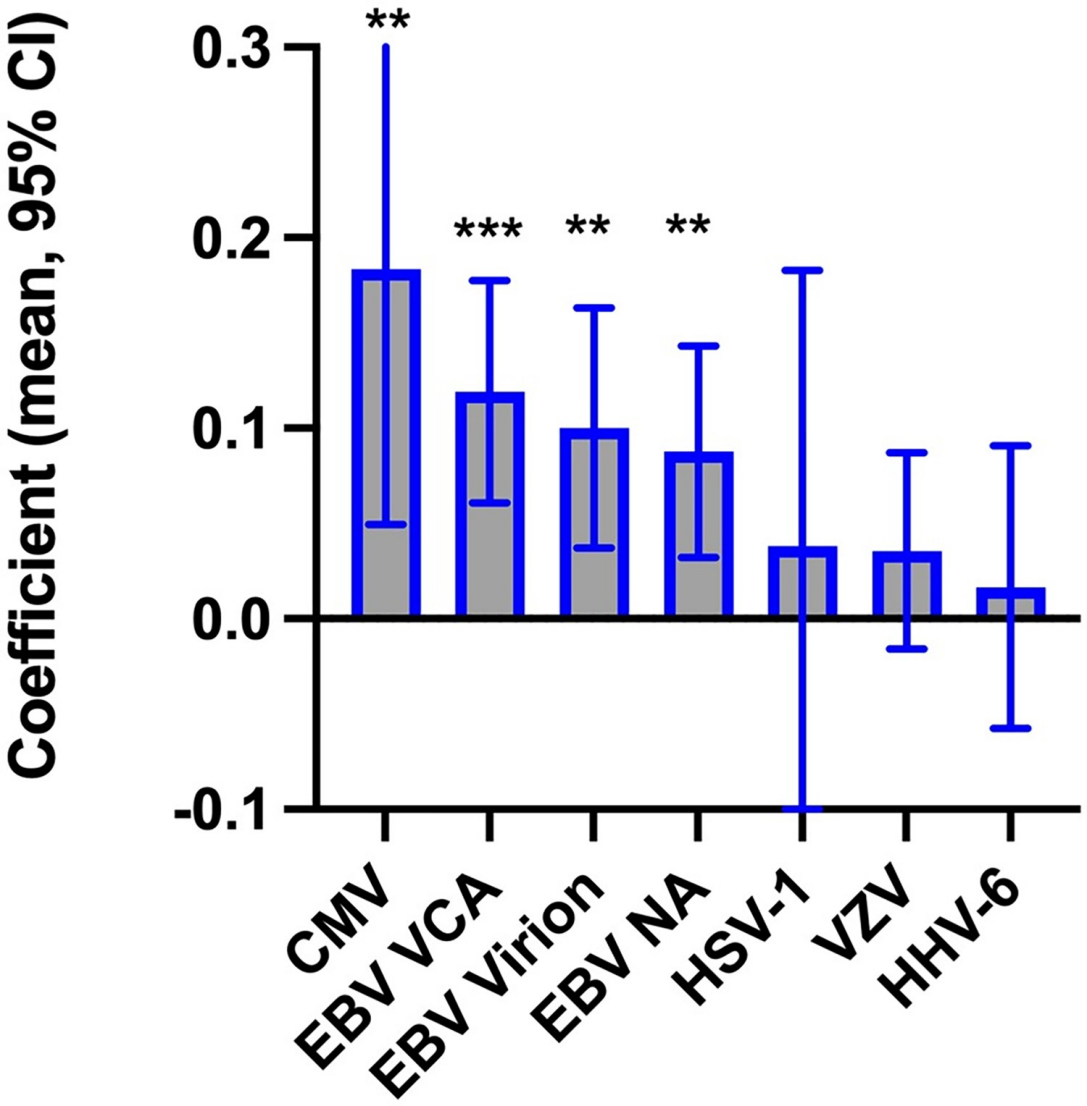
The association between cigarette smoking and quantitative levels of antibodies to Cytomegalovirus (CMV); Epstein Barr Virus nuclear antigen (EBV NA), virion (EBV virion), and anti-viral capsid antigen (EBV VCA); Herpes Simplex Virus Type 1 (HSV-1); Varicella Zoster Virus (VZV); and Human Herpes Virus 6 (HHV-6) from linear regression models employing age, sex, race, and psychiatric diagnosis as covariates; ** = p<.01, *** p<.001). N = 1323 except for HHV-6, N = 1206. (CMV- F(7, 1315) = 28.01, R^2^ = 0,1298, VIF = 1.32; EBV NA - F(7, 1315) = 21,82, R^2^ = 0,1041, VIF = 1.32; EBV Virion – F (7,1284) = 40.59, R^2^ = 0.1812, VIF = 1.32; EBV VCA F(7,1296) = 37.96, R^2^ = 0.1701, VIF = 1.32; HSV-1- F(7, 1315) = 11.35, R^2^ = 0.0570, VIF = 1.32; VZV – F(7, 1300) = 5.04, R^2^ = 0.0264, VIF = 1.31); HHV-6 – F(7,1198), R = 0.0182, VIF = 1.29).

The coefficients relating antibody levels to smoking status are depicted in **Fig 1**. Current smoking was significantly associated with levels of IgG antibody to three distinct EBV proteins: EBV NA (coefficient .088, 95% CI .032, .143, p = .002, q<.014), EBV Virion (coefficient .100, 95% CI .037, .163, p = .002, q<.014), and EBV VCA (coefficient .119, 95% CI .061, .177, p = .00004, q<.0016). The levels of IgG antibody to CMV were also significantly associated with current cigarette smoking (coefficient .183, 95% CI .049, .317, p<.001, q<.007). The levels of antibodies to HSV-1, HHV-6, and VZV were not significantly associated with current smoking status (all p>.05). Antibody levels were not significantly associated with the interaction variable calculated between diagnosis and cigarette smoking (all p>.05), indicating that the relative relationship between smoking and the antibody levels was not dependent upon clinical diagnosis.

**Fig 2:**
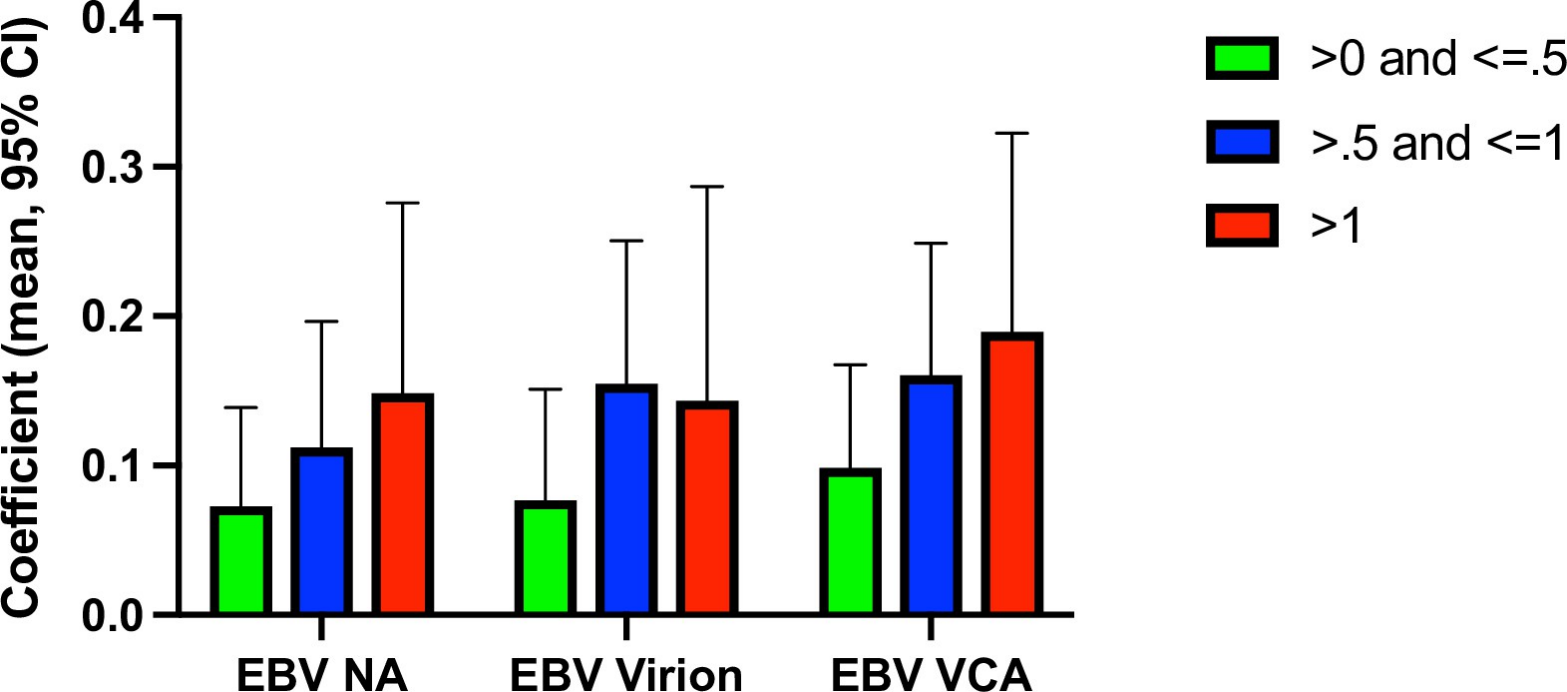
The association between packs per day (ppd) smoked and antibodies to Epstein Barr Virus nuclear antigen (EBV NA), virion (EBV virion), and anti-viral capsid antigen (EBV VCA) from linear regression models employing age, sex, race, and psychiatric diagnosis as covariates. (CMV- F(9, 1299) = 28.01, R^2^ = 0.1314, VIF = 1.28; EBV NA - F(9, 1299) = 16.97, R^2^ = 0.1052. VIF = 1.28; EBV Virion – F (9,1269) = 31.71, R^2^ = 0.1836, VIF = 1.28; EBV VCA F(9,1280) = 29.82, R^2^ = 0.1733, VIF = 1.28).

In the case of the antibodies which were associated with cigarette smoking, we also examined the association between these quantitative levels and the amount of daily smoking as measured by the reported packs per day at the time of assessment as shown in **Fig 2**. The number of cigarettes smoked per day was associated with levels of IgG antibody to EBV VCA (p = 6 x 10^-6^, q<.001), EBV Virion (p = .0005, q<.002), and EBV NA (p = .0004, q = .0016). The relationship between IgG antibodies to CMV and was not statistically significant (q>.05).

**Fig 3:**
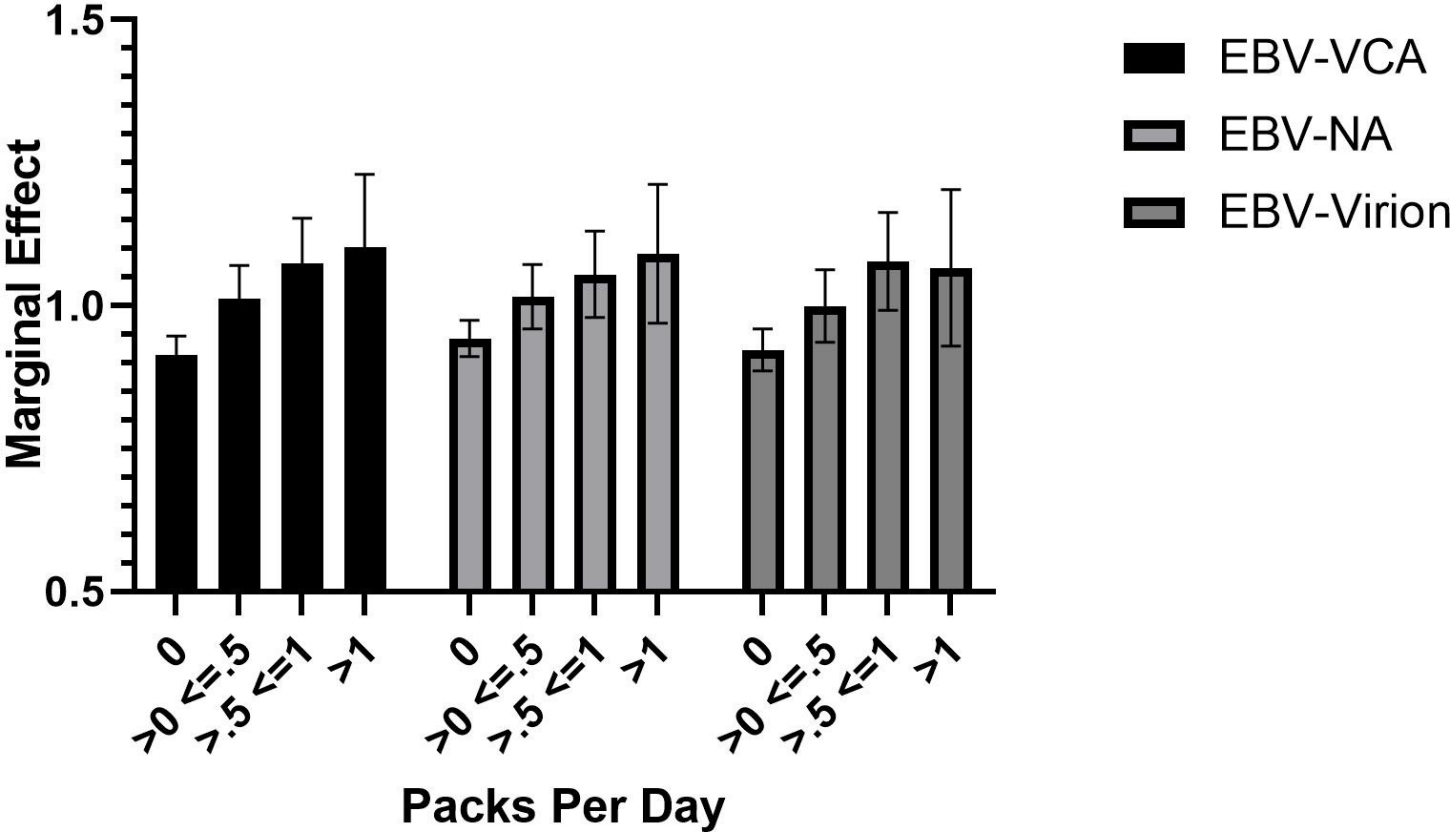
Marginal effects (mean +/- 95% confidence intervals of the indicated EBV antibody by number of cigarettes smoked per day adjusted for age, gender, race, and diagnostic group.

Further analysis of the marginal effects indicates an incremental increase in levels for all 3 EBV antibody measures related to the number of cigarettes smoked per day (**Fig 3**).

We examined the interaction between diagnosis and the presence or absence of cigarette smoking by including an interaction term in the regression models. We found that individuals with Major Depressive Disorder had lower levels of smoking associated antibodies to CMV (coefficient = -.74, 95% CI -1.33, -.143, p=<.02). Other interactions between diagnostic group and cigarette smoking or cigarette packs per day were not significant.

We explored the potential interaction between cigarette smoking and sex (or gender identity which in this set is identical) by the addition of this interaction term to the regression models exploring the relationship between antibody levels and smoking. None of the regression models showed significant evidence of this interaction (all p>.1). We also examined the potential interaction between cigarette smoking and level of parental education on the levels of antibodies again employing interaction terms in the regression models. None of the regression models showed significant evidence of this interaction (all p>.1).

The demographic and antibody data employed for the analyses are included in S1 Dataset.

## Discussion

Our study documents that the levels of antibodies to two Herpesviruses, EBV and CMV, are significantly increased in individuals who smoke cigarettes. This was the case for individuals diagnosed with a psychiatric disorder as well as individuals without a history of such a disorder. The relationship between antibody levels and smoking was independent of gender and SES with the latter measured by parental education.

In the case of EBV, antibodies were increased to three different sets of viral antigens and were proportionate to the number of cigarettes smoked per day. Initial infection with EBV generally occurs during adolescence, at which time latency is established in immune cells. The efficiency of primary infection and subsequent replication of Herpesviruses is controlled by a complex set of interactions between viral proteins and the host immune system. Viral replication is associated with increased levels of IgG antibodies to a range of viral proteins. The efficacy of the immune response and subsequent viral replication is partially under genetic control but also determined by a number of environmental factors such as diet and stress [13–15]. Our results suggest that cigarette smoking is an additional environmental factor which is associated r with increased levels of EBV antibodies. EBV has been linked to a number of disorders. For example, both EBV replication and cigarette smoking have been shown to be associated with nasopharyngeal carcinoma occurring largely in China [16]. EBV has also recently been associated with autoimmune disorders such as multiple sclerosis [17]. It is of note that cigarette smoking is also a risk factor for multiple sclerosis suggesting that the interaction between cigarette smoking and EBV should also be evaluated in the etiology of this disorder [18]. The association between EBV antibody levels and smoking is further supported by a recent Mendelian randomization study [17].

We also found increased levels of antibodies to CMV in individuals who smoked cigarettes. CMV infections usually first occur in infancy r childhood, and latency leads to lifelong viral persistence. The dynamics of CMV infection, latency, and reactivation are less well understood than those involving EBV but are likely to also involve complex interactions with the host immune system. CMV infections are well recognized as causes of acute infection in immunocompromised individuals and neonates [19, 20]. CMV has been linked to a number of chronic disorders in immunocompetent individuals including brain tumors, atherosclerosis, cognitive decline and accelerated aging, possibly related to telomere shortening [21]. Our findings indicate that cigarette smoking may be a contributory factor in the relationship between CMV and these chronic somatic disorders. Longitudinal studies will be necessary to better define the relationship between smoking, antibody levels, and subsequent consequence of infection.

It is of note that, while we found increased levels of antibodies to EBV and CMV in individuals who smoked cigarettes, we did not find such increases in the levels of antibodies to other human Herpesviruses such as HSV-1, VZV, or HHV-6. There are similarities among the human Herpesviruses in terms of viral structure and modes of transmission. However, there are substantial differences in the composition and organization of the viral genomes and transcribed proteins as well as in the interactions with the host immune system [22]. The specific pathways by which cigarette smoking may lead to increases in the levels of antibodies to some Herpesviruses but not others should be the subject of future investigations. Furthermore, the differences among the Herpesviruses suggest that our findings are not simply related to increased viral exposure among smokers since such exposures would be expected to be similar for the Herpesviruses evaluated in this study.

The finding of increased levels of antibodies to EBV and CMV but not the other herpesviruses in individuals who smoke is of interest. In the case of EBV, this virus has recently been shown to replicate and establish persistence in nasopharyngeal epithelial cells as well as B-cells [23]. Similarly the lungs have been suggested as a possible site for CMV latency and reactivation [24]. The health consequences of the interaction between cigarette smoke viruses within the nasopharynx and lungs should be the subject of future investigations.

It is also of note that increases in antibody levels to EBV and CMV were found both in cigarette smokers with serious mental illness and comparison individuals without a psychiatric disorder. It is also of note, however, that individuals with schizophrenia and other serious mental illnesses have a high prevalence of cigarette smoking and smoking-related disorders. Therefore, although our findings were not associated with a specific diagnosis, they do not preclude that EBV and CMV may also contribute to comorbid chronic disorders which are particularly prevalent in individuals with serious mental illness. Of interest in this regard would be cardiac, pulmonary, and cognitive disorders since they are particularly damaging in this population.

Limitations of the study include that no information was available about other potential confounding factors which may be associated with Herpesvirus IgG levels such as body mass index, alcohol consumption, and other medical conditions.

## Supporting information

S1 Table Antibody measurements. (DOCX)

S1 Dataset Minimal dataset for study. (XLS)
