# Supplementary material for: Cigarette smoking is associated with Herpesviruses in persons with and without serious mental illness
Source: PLoS One. 2023 Jan 18;18(1):e0280443. doi: 10.1371/journal.pone.0280443 (PMC9847975; doi:10.1371/journal.pone.0280443)
Supplement: S1 Table — (DOCX) [file pone.0280443.s001.docx]

Supplemental Table 1. Raw Optical Density Values for study population

Variable | Obs Mean Std. Dev. Min Max

-------------+---------------------------------------------------------

cmvigg_m | 1,323 .994142 1.155157 .012 4

ebvigg_m | 1,323 1.00361 .5749671 .021 3.017

ebv_gf_od_m | 1,292 1.10905 .7083555 .075 3.325

ebv_vcaigg~m | 1,304 1.519939 .8452399 .01 4

hsv1igg_m | 1,323 .972777 1.187324 .031 4

-------------+---------------------------------------------------------

hsv2igg_m | 1,323 .3894467 .8071061 .029 4

vzvigg_m | 1,308 1.515482 .8347248 .046 4

hhv6igg_m | 1,206 1.207152 .7876888 .042 3.727
